# Evaluation of data-driven respiratory gating waveforms for clinical PET imaging

**DOI:** 10.1186/s13550-018-0470-9

**Published:** 2019-01-03

**Authors:** Matthew D. Walker, Andrew J. Morgan, Kevin M. Bradley, Daniel R. McGowan

**Affiliations:** 1 Radiation Physics and Protection, Churchill Hospital, Oxford University Hospitals NHS Foundation Trust, Oxford, OX3 7LE UK; 2 Department of Radiology, Churchill Hospital, Oxford University Hospitals NHS Foundation Trust, Oxford, UK; 3 Department of Oncology, Old Road Campus Research Building, University of Oxford, Oxford, UK

**Keywords:** PET/CT, Motion, Respiratory gating, Data-driven gating, Imaging

## Abstract

**Background:**

We aimed to evaluate the clinical robustness of a commercially developed data-driven respiratory gating algorithm based on principal component analysis, for use in routine PET imaging.

**Methods:**

One hundred fifty-seven adult FDG PET examinations comprising a total of 1149 acquired bed positions were used for the assessment. These data are representative of FDG scans currently performed at our institution. Data were acquired for 4 min/bed position (3 min/bed for legs). The data-driven gating (DDG) algorithm was applied to each bed position, including those where minimal respiratory motion was expected. The algorithm provided a signal-to-noise measure of respiratory-like frequencies within the data, denoted as *R*. Qualitative evaluation was performed by visual examination of the waveforms, with each waveform scored on a 3-point scale by two readers and then averaged (score *S* of 0 = no respiratory signal, 1 = some respiratory-like signal but indeterminate, 2 = acceptable signal considered to be respiratory). Images were reconstructed using quiescent period gating and compared with non-gated images reconstructed with a matched number of coincidences. If present, the SUV_max_ of a well-defined lesion in the thorax or abdomen was measured and compared between the two reconstructions.

**Results:**

There was a strong (*r* = 0.86) and significant correlation between *R* and scores *S*. Eighty-six percent of waveforms with *R* ≥ 15 were scored as acceptable for respiratory gating. On average, there were 1.2 bed positions per patient examination with *R* ≥ 15. Waveforms with high *R* and *S* were found to originate from bed positions corresponding to the thorax and abdomen: 90% of waveforms with *R* ≥ 15 had bed centres in the range 5.6 cm superior to 27 cm inferior from the dome of the liver. For regions where respiratory motion was expected to be minimal, *R* tended to be < 6 and *S* tended to be 0. The use of DDG significantly increased the SUV_max_ of focal lesions, by an average of 11% when considering lesions in bed positions with *R* ≥ 15.

**Conclusions:**

The majority of waveforms with high *R* corresponded to the part of the patient where respiratory motion was expected. The waveforms were deemed suitable for respiratory gating when assessed visually, and when used were found to increase SUV_max_ in focal lesions.

## Background

Respiratory motion during PET data acquisition is unavoidable and often degrades clinical image quality. Features within the abdomen and thorax are generally blurred in the cranio-caudal direction when respiratory motion is left unaccounted for in the PET image reconstruction. A range of image artefacts can also occur. These are mostly due to a mismatch between the CT attenuation correction image, which is typically a snapshot at a single phase of respiration, and the PET image which typically represents an average over the whole respiratory cycle [1–3].

Many approaches have been demonstrated to mitigate the degrading effects of respiratory motion in PET-CT. For both the PET and CT modalities, methods fall into one of two categories. The first of these is instructed breathing, such as breath hold or repeated breath hold techniques. The second category is tracking of the respiratory phase, sometimes with gated exposure (for CT), and with compensated image processing (e.g. respiratory gated image reconstruction). The short exposure time (seconds) required in CT makes breath hold techniques compatible with this modality, and they are frequently used clinically. Repeated breath-hold PET has been performed [4, 5] but is not common, due to the much longer duration (minutes) required for PET data acquisition. Tracking the respiratory cycle by means of an external system, such as a pressure belt or a camera, is commonly performed for both CT and PET. Commercial products that interface with the PET-CT scanner include the respiratory gating system AZ-733VI (Anzai Medical; Tokyo, Japan) and the Real-time Position Management™ (RPM) Respiratory Gating system (Varian Medical Systems; CA, USA). While these external systems do provide respiratory gating solutions, they also require time to set up on the patient and occasionally do not yield a useful gating waveform [6]. Rather than using an external system to track the respiratory cycle, it is also possible to extract a respiratory signal from the PET data itself in what is known as data-driven gating (DDG). The periodic motion of the radioactivity within the patient, attributable to respiration, can be extracted from either the PET raw data (e.g. from a time series of short-duration sinograms [7]) or from PET images (e.g. from a time series of short-duration PET images [8]). In both cases, the duration must be a small fraction of the respiratory period, e.g. 0.5 s. Data-driven gating has several potential advantages as compared to tracking the respiratory cycle using an external system. DDG is based on the motion of the radioactivity within the patient and hence directly linked to the respiratory motion of organs and tumours. External devices generally track the position of the chest wall and assume that organs of interest move synchronous to this. In fact, organs and lesions within the body can exhibit respiratory motions that are phase-shifted compared to the chest wall motion [9]. There is no set-up time involved for DDG, as no external system or tool is attached to the patient. The DDG waveforms are inherently time-synchronised to the scanner without the possibility of a time offset, which could otherwise prevent robust gating. The clinical impact and feasibility of using DDG have been investigated by Kesner et al. [10]. Until now, however, commercial data-driven respiratory gating solutions have not been widely available.

In this study, we evaluated respiratory waveforms generated by a DDG algorithm which has been commercially developed by GE Healthcare (Waukesha, WI, USA), marketed with the name *MotionFree*, and which has the 510(k) approval for use in the USA. The algorithm applies principal component analysis to a time series of down-sampled sinograms, with one of these components then selected and assumed to capture the period of the respiratory motion. The variation of the weighting factor for this component over time provides a respiratory waveform similar to that provided by the external respiratory gating equipment. The method has been recently validated in phantom studies, and several patient examples have been presented [11, 12]. The commercial implementation is built on the work of Thielmans et al. [7, 13, 14]. A preliminary version of this work has been presented previously [15].

## Methods

### Study design

This evaluation of the prototype DDG algorithm focused on several technical aspects of the respiratory gating to allow practical implementation in routine clinical use. The first part of the evaluation concerned the quality of the respiratory waveforms generated by the algorithm, as assessed on 157 FDG PET-CT scans and in an experiment using a moving phantom. The algorithm generates its own metric of waveform quality (based on a signal-to-noise ratio and denoted as *R*). We first confirmed, using the phantom data, how *R* increases when the amplitude of the respiratory motion applied to the phantom increases. Following this, the DDG waveform from each bed position of the 157 patient examinations was visually examined and scored. These scores were compared to the algorithm’s own metric. The variation of these scores with the location of the PET bed position, i.e. the patient’s body part, was investigated.

The next step in the evaluation was an assessment of the magnitude of changes in SUV_max_ values for well-defined lesions in the thorax or abdomen. Reconstruction was performed using quiescent period gating (retaining 50% of coincidences) [16] and compared to reconstruction without respiratory gating.

Finally, we examined the dependence of acquisition duration on the algorithm’s metric of waveform quality. Part way through a patient acquisition, the algorithm makes an assessment of the magnitude of respiratory motion based on this metric, from which it may trigger (above some threshold) an extension to the acquisition duration for the current bed position. This automatic extension of the scanning duration for bed positions where respiratory motion is detected is expected to be useful, as it allows the application of quiescent period gating for these bed positions while maintaining an adequate number of counts in the retained dataset. When the magnitude of the respiratory motion, as inferred from the quality metric, is below the threshold, it is assumed that there is little benefit and hence no need for respiratory gating. The scan duration is then not extended, and a non-gated image will be generated making use of all the acquired coincidences; patient throughput is hence improved while image quality is retained. In combination, our evaluation aimed to enable a threshold value of *R* to be selected based on the expected veracity of the waveform.

### Phantom data

Data were acquired from a moving phantom and processed using the DDG algorithm. The phantom setup consisted of a foam phantom containing five small ^68^Ge spheres (the VQC-068 phantom; Eckert & Ziegler; Valencia, CA, USA), each with an activity of 13 kBq. This phantom was placed on the QUASAR™ respiratory motion platform (Modus QA; London, ON, Canada) that performed periodic motions in the Z direction, using a driving waveform that was typical for normal respiration (named Typical1, supplied by the manufacturer). A cylindrical, uniform ^68^Ge phantom (20 cm diameter, 19 cm height, activity concentration of 0.2 kBq/mL) was placed on the scanner couch, adjacent to the platform to provide a source of activity outside the scanner’s coincidence field-of-view. This setup was chosen to represent the case of imaging lesions within the lung. Data were acquired with the platform driving the phantom with a range of amplitudes (0–15 mm, corresponding to maximum displacements of 0–30 mm). There were three repeated acquisitions at each amplitude. Each acquisition was at the same bed position and was 180 s in duration. Each acquisition was processed with the full 180-s duration, and also after being split into two frames of 90 s and four frames of 45 s. This provided between 3 and 12 *R* values at each amplitude for the given frame duration, from which the mean and standard deviations were found. The standard error on the mean was calculated as the standard deviation divided by √*n*, where *n* equalled 3 for 180 s, 6 for 90 s and 12 for 45 s.

### Patient data

One hundred fifty-seven sets of adult [^18^F]FDG PET examinations comprising a total of 1149 acquired bed positions were used to evaluate the performance of the data-driven gating algorithm. These scans were acquired on 20 different days in December 2017 and January 2018, randomly chosen and are representative of [^18^F]FDG scans currently performed at our institution. Patients fasted for more than 6 h prior to i.v. administration of 4 MBq/kg [^18^F]FDG. The uptake period was 90 min. The PET-CT examination commenced with a free-breathing helical CT, followed by free-breathing PET with 4 min/bed position (3 min/bed for legs). The PET-CT scanner used was a Discovery 710 (GE Healthcare; Waukesha, WI, USA). This is a fully 3D PET-CT scanner incorporating a time-of-flight technology with LYSO-based scintillation detectors [17].

### DDG waveform generation and assessment

The data-driven gating algorithm [13] was applied to each bed position, including those where minimal respiratory motion was expected. The algorithm performs principal component analysis on a time series of sinograms to identify the particular sinogram elements that change during the scan. The weighting factor for each principal component describes how that component changes with time and may capture the phase and period of respiratory motion. The algorithm first bins listmode data into a set of down-sampled sinograms. Each sinogram in this set is 0.5 s in duration and downsampled in its radial and angular components. No downsampling is applied in the axial components to retain maximum spatial resolution in the direction most commonly associated with changes caused by respiratory motion. Time-of-flight information is discarded in this implementation. There is then a correction for low-frequency shifts in the total counts, to account for changes that are not due to respiration (e.g. those from radiotracer redistribution). The sinograms are then masked using a threshold to remove those parts of the sinogram that are considered to be outside the patient. PCA is then applied, and the first three principal components assessed for the strength of the respiratory signal that they contain. This assessment is performed using a signal-to-noise measure of respiratory-like frequencies within the data, denoted as *R*, derived from the power spectrum of the transformed dataset. The maximum value of the Fourier transform of the eigenvector’s weight function within the frequency range for respiration (0.1–0.4 Hz) is divided by its mean value for frequencies above this range (0.4–1.0 Hz). From these three ratios (respiratory maximum/mean noise), the largest one is chosen and defined as *R*. The corresponding principal component becomes the respiratory principal component, with the respiratory waveform extracted using its eigenvector. The respiratory waveform is thus created from this weighting factor, up-sampled to 0.25-s intervals using linear interpolation.

For each waveform, a qualitative evaluation was also performed. Each auto-scaled waveform was reviewed by two clinical scientists (medical physicists) blinded to the *R* value and scored on a 3-point scale assessing its suitability for clinical use (score *S* of 0 = no respiratory signal, 1 = some respiratory-like signal but indeterminate, 2 = acceptable signal considered to be respiratory). The scores from the two readers were averaged. The axial location of the centre of each bed position, and hence each corresponding *R* and *S* values, was determined relative to the reference location at the most superior point of the liver as determined from the CT image.

We expected *R* to have some dependence on the acquisition duration, and any threshold applied based on *R* may hence need to be scan duration-dependent. As such, all the patient data from bed positions with a 4-min acquisition time were re-processed and the *R* values re-calculated for a range of acquisition durations (10–240 s), keeping an initial portion of the data only. The data were analysed by linear regression of the reduced duration *R* values with those from the reference time of 4 min (240 s). The slope of the linear fit (intercept fixed at 0) was used as a measure of the systematic change in *R* with duration. The mean values of *R* were also calculated, considering only bed positions with *R* ≥ 15 as found with the full 240-s bed duration.

### Assessment of DDG-reconstructed images

PET images were reconstructed using DDG-triggered quiescent period gating (QPG), retaining 50% of coincidences [16]. Images were also reconstructed without gating, using data from the first 2 min of each bed position, such that the number of coincidences used for image reconstruction was matched to that used for the gated image. A non-gated image was also reconstructed using the full 4 min of data at each bed position. The reconstruction algorithm was the manufacturer’s Bayesian penalised likelihood reconstruction (Q.Clear) with a beta value of 400 [18].

The three [^18^F]FDG PET-CT images for each patient were reviewed by a clinical scientist (medical physicist) and screened for the presence of a focal, avid lesion in the upper abdomen or thorax (including the rib cage). When multiple lesions were present, the most focal was chosen for analysis. The SUV_max_ from the lesion was measured on each PET image. The axial location of the lesion was also recorded to allow the *R* value for this lesion to be identified from its bed position. In the case of a lesion being identified in the overlap region of two bed positions, the assigned values of *R* were calculated as the sensitivity-weighted average of the two values. This allowed exploration of the relationships between the lesion locations, *R* values and changes in SUV_max_ due to the application of DDG.

## Results

**Fig. 1:**
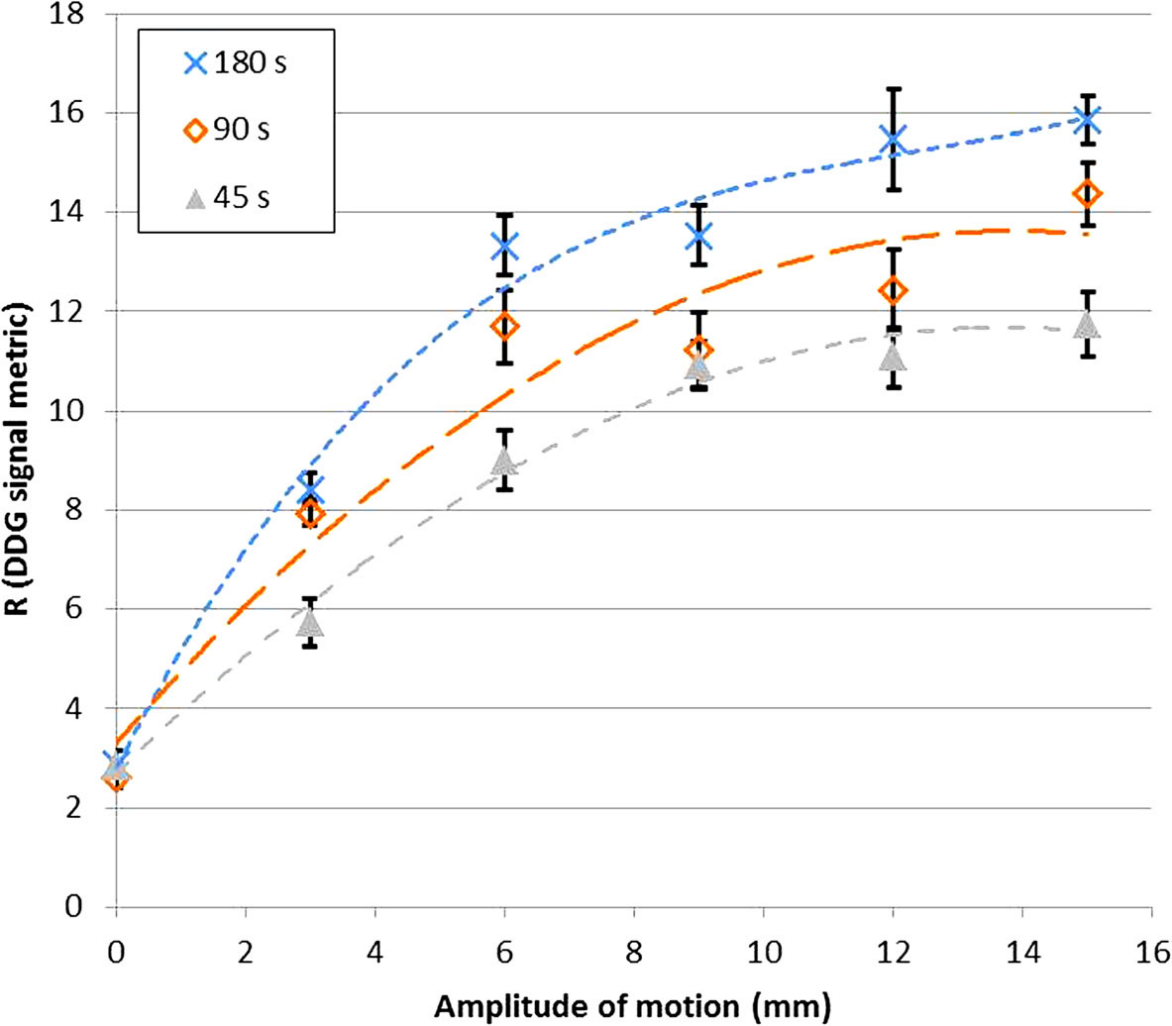
Relationship between *R* and motion amplitude found for a phantom performing respiratory-like motions. The maximum displacement was 30 mm, corresponding to a 15-mm amplitude. Lines of best fit are shown (using third-order polynomials). Results are presented for scan durations equal to 180 s, 90 s and 45 s. Error bars represent standard errors on the mean

The phantom experiment confirmed that increasing the amplitude of respiratory-like motion led to an increase in *R*. The data also demonstrated a reduction in *R* for shorter acquisition durations. These results are presented in Fig. 1.

**Fig. 2:**
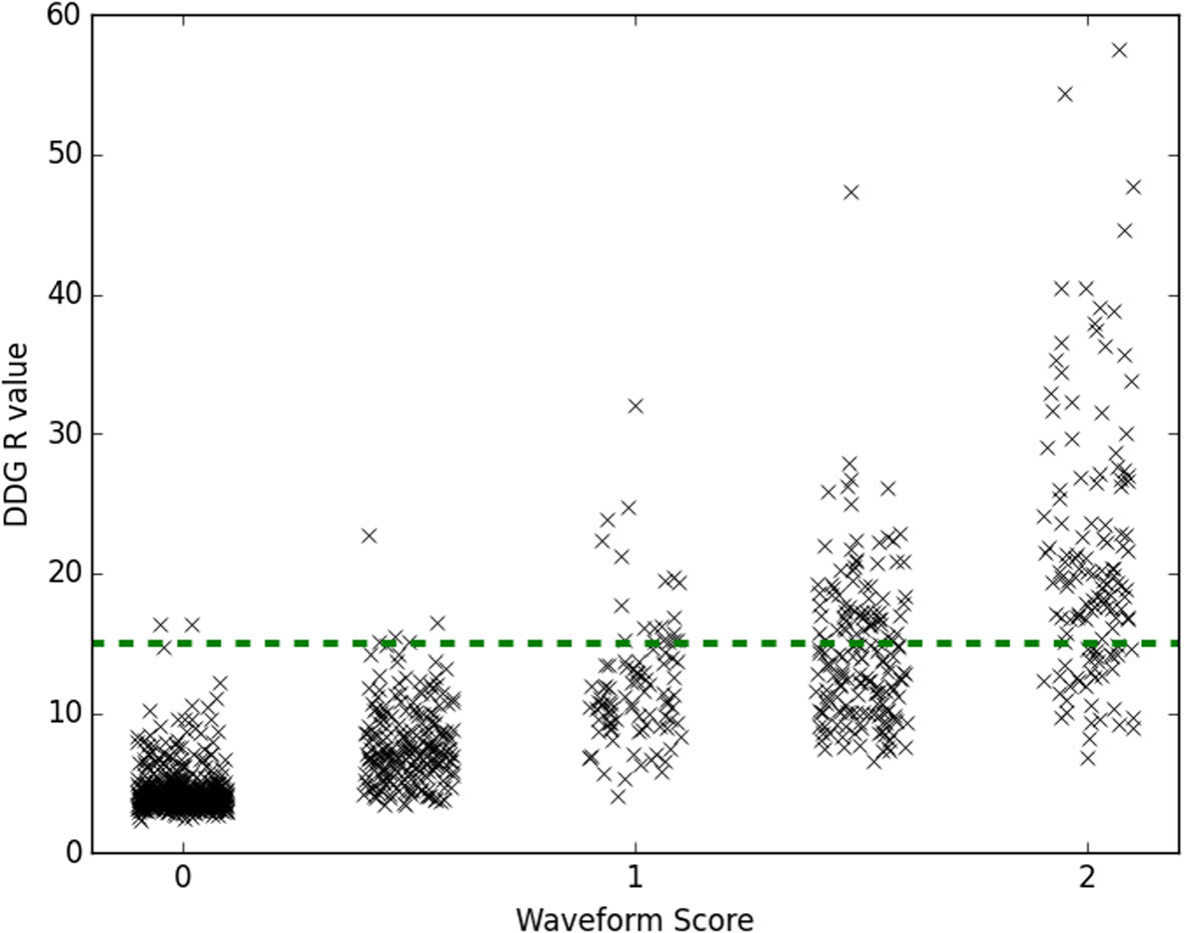
Relationship between the algorithm’s figure of merit for the waveform (the *R* value) and the waveform score *S* as determined by two readers from visual inspection of the waveform. The horizontal dashed line shows a threshold, *R* = 15

**Fig. 3:**
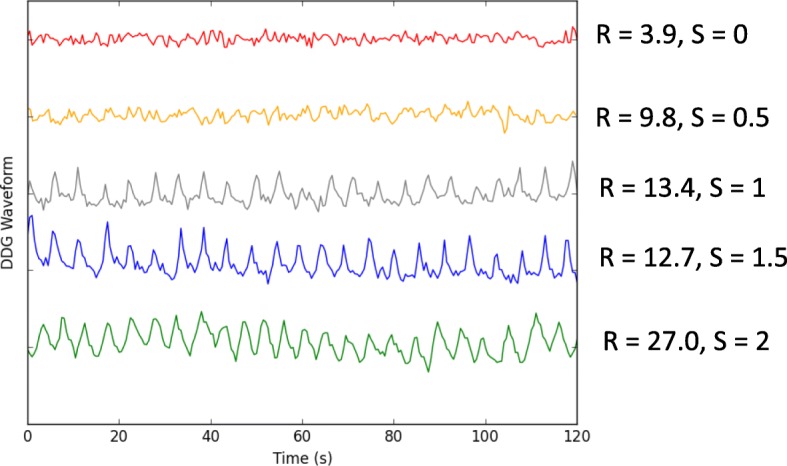
Example waveforms showing the first 120 s (out of 240 s) of data for five different bed positions. The *R* values and mean scores *S* are shown for each waveform

From the patient data with 1149 bed positions, there was a strong (*r* = 0.86) and statistically significant (Wald test, *p* < 0.001) positive correlation between the natural logarithm of the algorithm’s figure of merit for the waveform, log(*R*) and the score *S* assigned from visual inspection. The distribution of *R* values for different scores *S* is shown in Fig. 2, where the good agreement between the two measures can be observed. There were 2 cases (out of 1149; 0.2%) where *S* equalled 0 while *R* was greater than 15. There were 35 bed positions (3%) where waveforms were scored as 2 while *R* was less than 15. Examples of waveforms for a range of scores *S* and *R* values are provided in Fig. 3.

**Fig. 4:**
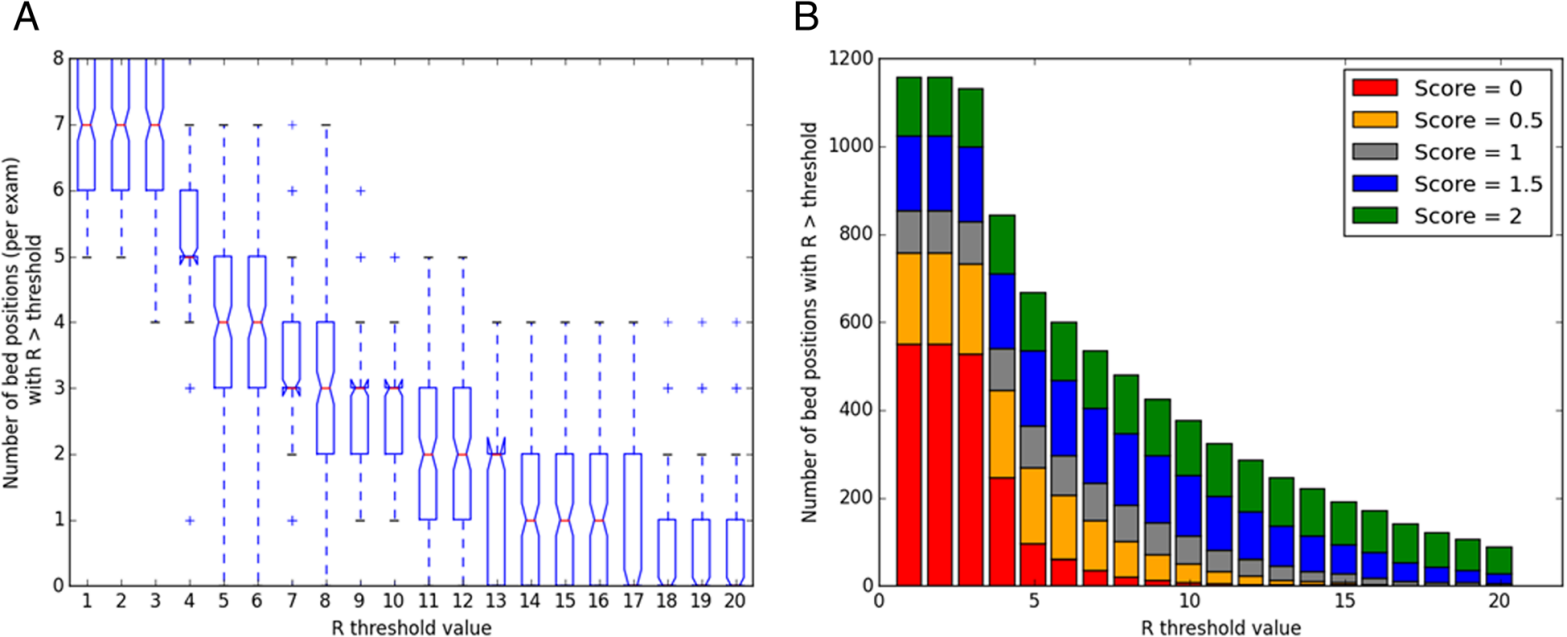
**a** Number of bed positions per patient with *R* greater than a set threshold, shown for *R* value thresholds between 1 and 20. **b** Total number of bed positions with *R* greater than the *R* value threshold, split by the score *S* assigned to the waveform from visual inspection of all bed positions in 157 examinations. Shown for *R* value thresholds between 1 and 20. For data in both figures, the acquisition duration per bed position was 240 s (4 min)

For a practical clinical implementation, one could set a threshold on the *R* value, above which respiratory gating is applied. As the chosen threshold is increased, the number of bed positions with *R* exceeding the threshold reduces. This is demonstrated in Fig. 4a, which also shows that there was an average of 1.2 bed positions per patient examination that had an *R* value exceeding the default threshold of 15. Figure 4b shows that as the *R* value threshold increases, a higher proportion of waveforms is scored as visually acceptable. Of the 191 bed positions with *R* greater than the threshold of 15, 164 of them (86%) were scored as acceptable for respiratory gating (*S* ≥ 1.5).

**Fig. 5:**
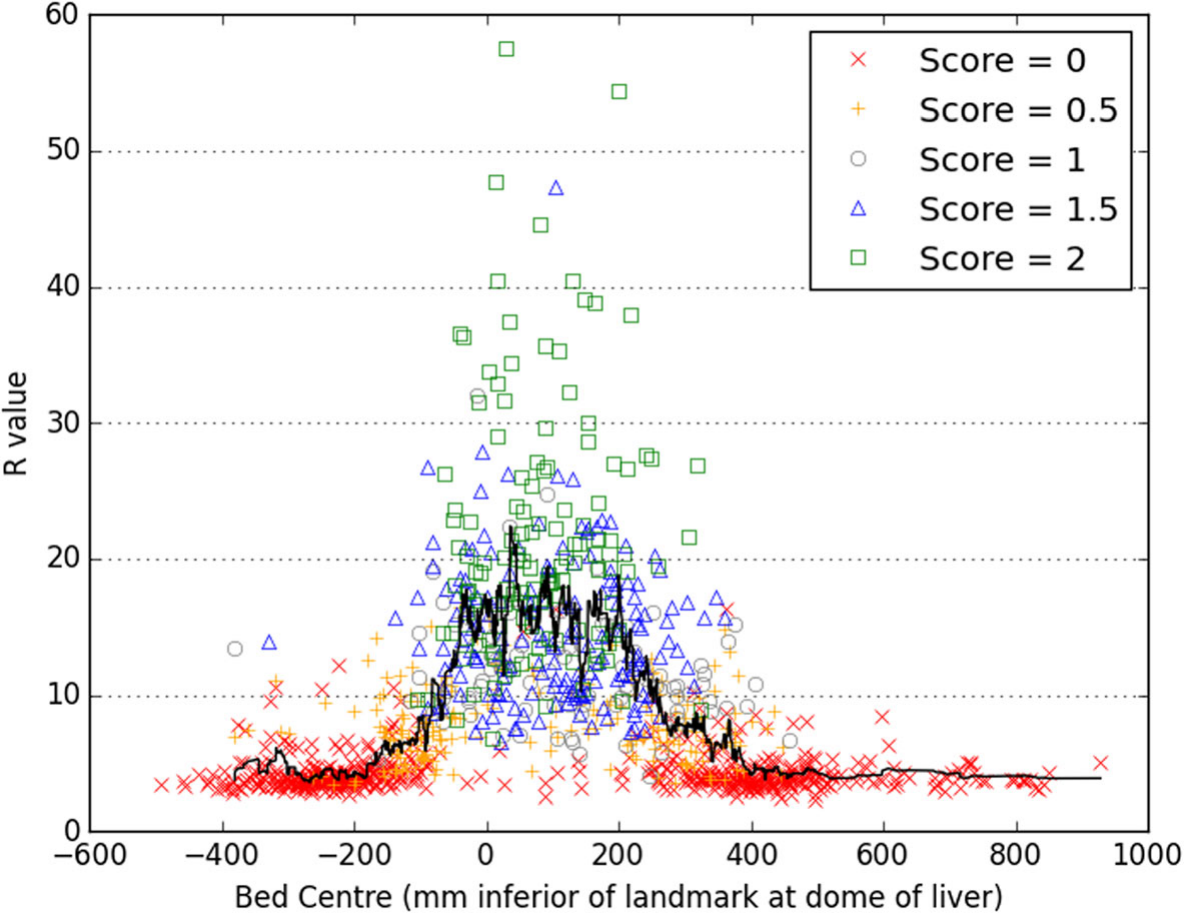
The *R* values and scores *S* for waveforms generated at the different bed positions for 157 patients. A moving average is shown as a solid black line. The most superior point of the liver determined from CT (i.e. the dome of the liver) is used as the reference level

Waveforms with high *R* and *S* were found to originate from bed positions corresponding to the thorax and abdomen: 90% of waveforms with *R* ≥ 15 had bed centres in the range 5.6 cm superior to 27 cm inferior from the dome of the liver. Using the eyes as an alternative reference point, this corresponded to 26–57 cm inferior of eye level. This is presented in Fig. 5. For regions where respiratory motion was expected to be minimal such as the head and legs, *R* tended to be < 6 and *S* tended to be 0.

**Fig. 6:**
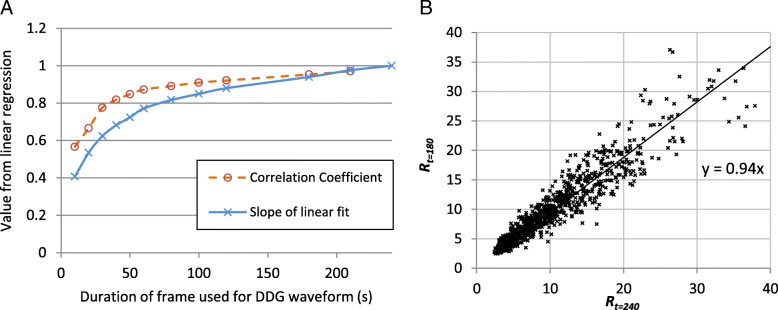
**a** Results from linear regression, comparing *R* calculated from a 240-s duration acquisition to *R* found for the first *t* seconds of the acquisition. **b** Example regression, showing *R*_*t* = 180_ compared to *R*_*t* = 240_

Analysis of the waveforms generated using reduced acquisition durations demonstrated that *R* is systematically lower for shorter acquisitions. This dependence of *R* on the acquisition duration is shown in Fig. 6a, where the slope from a linear regression (*R*_*t* = 240_ vs *R*_*t*_) is plotted for the various acquisition durations *t*. Pearson’s correlation coefficient is also displayed. A comparison of *R*_*t* = 180_ and *R*_*t =* 240_ is presented in Fig. 6b. Mean *R* values for waveforms with high *R* (*R*_*t* = 240_ ≥ 15) are provided in Table 1 and confirm a systematic dependence, which led to substantial reductions in *R* for short durations and which was statistically significant for all the durations tested.

**Table 1:** Mean *R* values from all bed positions that satisfied the condition *R*_*t* = 240_ ≥ 15

Scan duration (s)	10	20	30	40	50	60	80	100	120	180	210	240
Mean *R* [for *R*_*t* = 240_ ≥ 15]	7.8	10.6	12.8	14.3	15.2	16.3	17.3	18.2	18.7	20.1	21.0	21.7

A systematic reduction in the *R* value is seen for when the acquisition duration is reduced. All of the mean values are significantly lower than the mean value from 240 s of data (paired *t* test; *p* < 0.01)

**Fig. 7:**
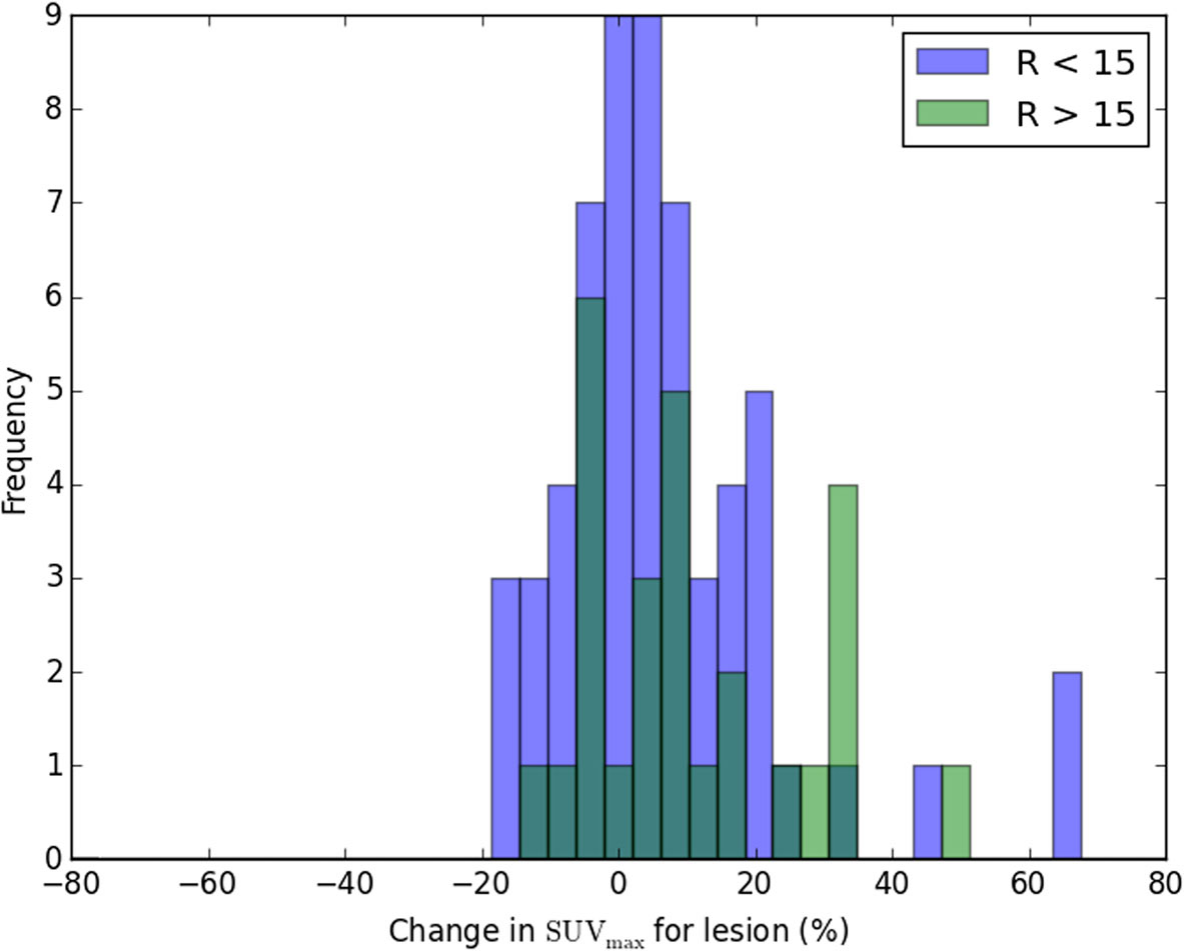
Histogram of change in SUV_max_. The data are split into two groups based on the corresponding *R* values, below and above an *R* value threshold of 15. Where data are overlaid, a darker colour bar is shown

Of the 157 examinations in this study, a focal lesion in the thorax or abdomen was found in 86 cases (with any *R* value). Application of data-driven QPG was found, on average, to increase the lesion’s SUV_max_ in comparison to non-gated images with a matched number of coincidences (*p* < 0.001; paired *t* test). In many cases, the change (either increase or decrease) was small and expected to be clinically insignificant. In other cases, there was a substantial increase that could be clinically significant. When only lesions with a corresponding *R* value greater than or equal to 15 were considered, the mean increase was 11%. Considering only those lesions with *R*_*t* = 240_ < 15, the mean increase was 6%. For all lesions regardless of the *R* value, the mean increase was 8%. The distribution of this change is presented as a histogram in Fig. 7. There was a small difference in SUV_max_ between the two sets of ungated images; those reconstructed from 2 min of data were, on average, 3% higher than those reconstructed from the full 4 min (*p* = 0.003; paired *t* test).

## Discussion

Several technical and practical aspects relating to a commercial implementation of data-driven gating have been evaluated. The algorithm’s figure of merit for the respiratory waveform signal, *R*, was found to be highly correlated with our assessments based on visual inspection. The phantom study confirmed that *R* increased with larger motion amplitudes. We also verified that *R* tended to be substantially higher when scanning a part of the patient where large respiratory motion was expected. These results support the use of an *R* value threshold to determine whether or not QPG should be applied.

The practicalities of such an approach led to several other questions that we addressed, including how *R* depends on the acquisition duration. This is of particular importance when considering live DDG, where an *R* value is determined part way through a bed position (typically around the completion time of the standard bed duration), with a decision then made based on the *R* value as to whether the acquisition should be automatically extended. QPG may then be applied while ensuring that the gated images have a sufficient number of counts. While this may be a seamless approach to the management of respiratory motion, the *R* value threshold needs to be chosen with care. It may be possible to devise improved measures of the suitability of DDG waveforms for respiratory gating, as well as the benefit that may be expected from applying such gating. Different metrics which have some similarity to *R* have been used in the evaluation of other DDG algorithms [19, 20]. The phantom study confirmed that larger motion amplitudes do lead to higher *R* values but also showed that the increase was not linear. When automating the decision of whether or not to apply DDG, it may be beneficial to assess both the motion amplitude alongside the validity of the DDG waveform. The *R* metric as currently implemented is sensitive to both, but is primarily designed as an indicator of the waveform validity, and is not a direct measure of the respiratory motion amplitude.

Another practical issue is that of patient throughput, which will be directly influenced by the choice of threshold in the case of live DDG. When all bed positions are screened for motion, we found an average of 1.2 bed positions per patient with *R*_*t* = 240_ ≥ 15. It is hence possible to calculate the expected additional scanning time needed when moving from a workflow without respiratory gating to one where live DDG is applied. On the other hand, if the current workflow made use of an external gating system and extended acquisition durations for 2 fixed bed positions, changing to live DDG may result in increased throughput.

The commercial release of a data-driven gating solution for respiratory motion represents an important step forward for the field. The development builds on many important contributions from various groups. The core component of all DDG algorithms is the method used to identify and thus extract a respiratory signal from the data. The intuitive image-based method tested by Bundschuh et al. [8] was to position a volume of interest (VOI) over the lesion and to then calculate the *z*-coordinate of the VOI’s centre of mass for each short time frame. The method was found to yield usable data for DDG, but it relies on a manual VOI definition and also requires the often time-consuming reconstruction of many image frames. An automated image-based method was developed by Kesner et al. [20], whose algorithm selectively incorporates image voxels to build a high-quality respiratory signal from their combined time-activity curves. Approaches based on the raw coincidence (sinogram) data, with no requirement for a dynamic image reconstruction, include the geometric sensitivity method [21] that utilises the fact that 3D PET scanners exhibit large variations in sensitivity with axial position. Periodic fluctuations in the coincidence counting rates may then be attributed to respiratory motion. This method was later developed further and compared to the waveform provided by the change in the axial centre of mass of the true coincidences [22]. The inclusion of time-of-flight information, as well as consideration of motion in other directions (e.g. anterior-posterior), was used in the centroid of the distribution algorithm recently evaluated by Ren et al. [23]. Kesner and Kuntner [24] developed a more complex, automated DDG algorithm that extracts data from selected pixels within the filtered projection data. Their algorithm combines several aspects of the aforementioned image-based works, incorporating only those sinogram pixels (projection bins) whose inclusion increases the standard deviation of the waveform as it is generated. On the other hand, the algorithm of Schleyer et al. uses spectral analysis to ascertain which sinogram pixels should be considered useful in the generation of the respiratory waveform [25]. Like the algorithm of Kesner and Kuntner [24], the automatic PCA-based DDG algorithm tested in this work involves extraction of a waveform from raw sinogram data with high temporal resolution but reduced spatial resolution [13]. Selection of appropriate pixels is however performed via principal component analysis. The temporal variations of the principal component weighting factors are then examined, and the component with the strongest respiratory frequency is retained. The variation of this component’s weighting factor with time is used as the DDG waveform.

Our evaluation has some limitations. The phantom experiment considered changes in *R* with motion magnitude but did so for an otherwise fixed radioactivity distribution and with relatively little activity in the scanner’s FOV. It is expected that *R*, and also the most appropriate *R* value threshold, depends to some degree on a variety of other factors in addition to the magnitude of the respiratory motion. These include the radioactivity distribution within the patient as well as the part of the patient currently being imaged. We do not recommend choosing an *R* value threshold based solely on the results of the phantom experiment shown in Fig. 1. Calculated *R* values could potentially be modulated by a number of factors including those that change the statistical quality of the data. *R* is hence expected to change not just with the region being imaged and scan duration but also the injected activity, uptake period, patient habitus, and especially if a different radiotracer is used. The appropriate threshold may hence be scanner and protocol dependent.

The current evaluation did not include a comprehensive clinical evaluation of the gated images nor was a comparison attempted against waveforms from an external device. Our data did however confirm that application of DDG led to an increased SUV_max_, on average. The observed increase due to respiratory gating was both expected and similar to that reported by others [26]. A comparison against the RPM device was made previously using anthropomorphic phantom data, for which the true driving waveform was known [12]. Although the similarities between waveforms from the PCA-DDG algorithm and the RPM system have been presented for several patients [7, 13], the benefit from such a comparison is limited by the lack of a gold standard. The reasonable agreement observed is reassuring, but it is difficult to ascertain the superiority of either method. There is a similar limitation in the current work, where neither the *R* value nor the score *S* from visual inspection can be considered as a gold standard for determining the validity of the waveform and its correspondence to the respiratory motion of internal organs. We anticipate that these important questions will be addressed by the results of a large, ongoing clinical study including detailed clinical evaluations.

## Conclusions

For the PCA-based DDG algorithm, the majority of waveforms with high *R* corresponded to the part of the patient where respiratory motion was expected, and the waveforms were deemed suitable for respiratory gating when assessed visually. Application of DDG was found to significantly increase the SUV_max_ of focal lesions by mitigating the blurring effects of respiratory motion.

## Abbreviations

DDG: Data-driven gating
PCA: Principal component analysis
QPG: Quiescent period gating
RPM: Real-time Position Management™ system
SUV: Standard uptake value
